# Baseline characteristics of people experiencing homelessness with a recent drug overdose in the PHOENIx pilot randomised controlled trial

**DOI:** 10.1186/s12954-023-00771-4

**Published:** 2023-04-04

**Authors:** Richard Lowrie, Andrew McPherson, Frances S. Mair, Kate Stock, Caitlin Jones, Donogh Maguire, Vibhu Paudyal, Clare Duncan, Becky Blair, Cian Lombard, Steven Ross, Fiona Hughes, Jane Moir, Ailsa Scott, Frank Reilly, Laura Sills, Jennifer Hislop, Natalia Farmer, Sharon Lucey, Stephen Wishart, George Provan, Roy Robertson, Andrea Williamson

**Affiliations:** 1 Pharmacy Services, Homeless Health/Research and Development, NHS Greater Glasgow and Clyde, Glasgow, G76 7AT Scotland, UK; 2 General Practice and Primary Care, School of Health and Wellbeing, College of Medical, Veterinary and Life Sciences, University of Glasgow, Glasgow, Scotland, UK; 3 Emergency Medicine, Glasgow Royal Infirmary, Glasgow, Scotland, UK; 4 School of Pharmacy, University of Birmingham, Birmingham, England, UK; 5 Addictions Psychiatry, NHS Ayrshire and Arran, Crosshouse, Scotland, UK; 6 Simon Community Scotland Street Team, Glasgow, Scotland, UK; 7 East End Addictions Services, Alcohol and Drug Recovery Service, Glasgow Health and Social Care Partnership, NHS Greater Glasgow and Clyde, Glasgow, UK; 8 Healthcare Improvement Scotland, Glasgow, Scotland, UK; 9 Department of Social work, School of Health and Life Sciences, Glasgow Caledonian University, Glasgow, UK; 10 Usher Institute, College of Medicine and Veterinary Medicine, The University of Edinburgh, Edinburgh, UK; 11 Shelter Scotland, Glasgow, UK

**Keywords:** Homelessness, Chronic disease, Opioid addiction, Primary health care, Randomised controlled trial, Public health, Polydrug use, Drug-related death

## Abstract

**Background:**

Drug-related deaths in Scotland are the highest in Europe. Half of all deaths in people experiencing homelessness are drug related, yet we know little about the unmet health needs of people experiencing homelessness with recent non-fatal overdose, limiting a tailored practice and policy response to a public health crisis.

**Methods:**

People experiencing homelessness with at least one non-fatal street drug overdose in the previous 6 months were recruited from 20 venues in Glasgow, Scotland, and randomised into PHOENIx plus usual care, or usual care. PHOENIx is a collaborative assertive outreach intervention by independent prescriber NHS Pharmacists and third sector homelessness workers, offering repeated integrated, holistic physical, mental and addictions health and social care support including prescribing. We describe comprehensive baseline characteristics of randomised participants.

**Results:**

One hundred and twenty-eight participants had a mean age of 42 years (SD 8.4); 71% male, homelessness for a median of 24 years (IQR 12–30). One hundred and eighteen (92%) lived in large, congregate city centre temporary accommodation. A quarter (25%) were not registered with a General Practitioner. Participants had overdosed a mean of 3.2 (SD 3.2) times in the preceding 6 months, using a median of 3 (IQR 2–4) non-prescription drugs concurrently: 112 (87.5%) street valium (benzodiazepine-type new psychoactive substances); 77 (60%) heroin; and 76 (59%) cocaine. Half (50%) were injecting, 50% into their groins. 90% were receiving care from Alcohol and Drug Recovery Services (ADRS), and in addition to using street drugs, 90% received opioid substitution therapy (OST), 10% diazepam for street valium use and one participant received heroin-assisted treatment. Participants had a mean of 2.2 (SD 1.3) mental health problems and 5.4 (SD 2.5) physical health problems; 50% received treatment for physical or mental health problems. Ninety-one per cent had at least one mental health problem; 66% had no specialist mental health support. Participants were frail (70%) or pre-frail (28%), with maximal levels of psychological distress, 44% received one or no daily meal, and 58% had previously attempted suicide.

**Conclusions:**

People at high risk of drug-related death continue to overdose repeatedly despite receiving OST. High levels of frailty, multimorbidity, unsuitable accommodation and unmet mental and physical health care needs require a reorientation of services informed by evidence of effectiveness and cost-effectiveness.

*Trial registration* UK Clinical Trials Registry identifier: ISRCTN 10585019.

**Supplementary Information:**

The online version contains supplementary material available at 10.1186/s12954-023-00771-4.

## Introduction

The individual, societal, health and economic burdens of homelessness and drug-related deaths are undisputed, overlap and are increasing [1–4]. People experiencing homelessness with problem drug use including opioid use disorder are at higher risk of fatal overdose than people with opioid use disorder living in mainstream society [5–7]. Polydrug use includes street benzodiazepines, and cocaine, for which there are no evidence-based substitute prescriptions known to prevent overdose [5, 8]. Almost half of all deaths among people experiencing homelessness are caused by polydrug overdose [2–4], suggesting an urgent need to investigate the characteristics including unmet needs of people experiencing homelessness with recent overdose, and test innovative, additional approaches to improving outcomes.

Experiencing a non-fatal overdose increases the odds of a subsequent fatal overdose [6, 11] and is associated with multiple physical and mental health problems [9, 10]. Multiple severe disadvantages including unmet mental health needs act synergistically to increase the risk of premature mortality from overdose and other causes [11–14]. Timely prevention and treatment of wide-ranging health problems in people with problem opiate use has been suggested as a way to prevent drug-related deaths [7, 12], but gold-standard randomised controlled trial evidence of health and housing interventions improving health outcomes is lacking [15–17]. In practice, accessing care for multiple problems requires attendance at different parts of a fragmented healthcare system where specialists cater separately for: problem drug use; mental health; physical health; housing; benefits; and social prescribing [18]. This suggests merit in testing accessible, holistic interventions [1, 19].

Helping people experiencing homelessness who have had an overdose requires a prior understanding of their detailed unmet health and social care needs. Previously, this understanding has come from studies describing secondary data e.g. using data linkage enabling inferences about populations [7, 12]. However, more nuanced data that capture information about non-prescribed and prescribed drug use, health service engagement, housing and other variables are also needed to understand unmet health and social care need at the individual level to inform targeted interventions [1, 20].

To date, published randomised controlled intervention trials targeting people experiencing homelessness with or without previous overdose (Additional file 1 provides a summary of recent randomised controlled trials (RCTs) lack sufficient detail on participants’ combined health and social care problems, treatments and management [15]. Multifaceted interventions aiming to improve health include peer health advisers, cash incentives or enhanced nurse led management of specific diseases, housing interventions and/or enhanced addictions management (Additional file 1) [15]. Randomised controlled trials in people with opioid use disorder (including people experiencing homelessness) have focussed on pharmacological interventions for opiate dependence in a younger cohort [21] than those experiencing homelessness with polydrug use [22]. To our knowledge, people experiencing homelessness post-overdose, despite their elevated risk of death, have not formed the target group of any intervention study (Additional file 1) [15].

In Scotland, 2021 was the first year since 2013 where drug misuse deaths have not increased (1330 in 2021 vs 1339 in 2020) [4]. This makes it the second highest annual total number of drug misuse deaths on record, 3.5 times higher than in the rest of the UK and many times higher than reports from European countries and per head of population than the USA [3, 4]. Deaths are caused, at least in part, by drugs other than opioids [4]. Strategic policy responses have prioritised uptake, access and patient choice in, substitute prescribing for opioid use disorder, provision of naloxone for emergency reversal of opioid overdose and heroin-assisted treatment for street heroin use [23]. In relation to problem street benzodiazepine use, clinical practice is informed by emerging evidence from a retrospective cohort study plus local guidance [24–27]. Detailed assessment and management of problem benzodiazepine use may or may not involve benzodiazepine prescribing.

It is not clear whether current approaches reduce overdoses or drug-related deaths in people with poly problem drug use.

Management approaches focussed on addressing problem drug use may not address patients’ competing priorities. These include unmet mental and physical health needs or a need for stable housing, which are associated with worse outcomes [14, 16, 17, 28, 29]. This suggests merit in offering holistic patient-centred care for those at highest risk of overdose, by addressing competing physical health problems, trauma and associated adverse childhood experiences [11, 30–32], housing and physical health problems.

PHOENIx (**P**harmacist and **H**omeless **O**utreach **E**ngagement and **N**on-medical **I**ndependent prescribing R**x**) is a collaborative NHS independent prescribing pharmacist and third sector homeless charity (Simon Community Scotland and Marie Trust) outreach intervention offering holistic health and social care support for people experiencing homelessness post overdose [33, 34]. We hypothesise that identifying and holistically addressing multiple health and social care problems in people experiencing homelessness may offer an alternative, successful route to reducing non-fatal and fatal overdoses.

This pilot study describes baseline findings from an ongoing pilot RCT. It fills gaps in our understanding of contemporary, comprehensive patient level health and social care needs, and tailored interventions aiming to improve outcomes in people experiencing homelessness. As a pilot RCT of a complex intervention, it follows a previous feasibility study [33] and precedes a planned definitive RCT conditional on achievement of progression criteria and a signal of improved patient outcomes [35]. We report baseline findings from the ongoing PHOENIx after overdose pilot randomised controlled trial, the results of which will be available in April 2023.

## Methods

This was a prospective, parallel group, randomised controlled pilot study.

### Participants

Participant eligibility criteria are described in Table 1 and have been described in detail previously [36].

**Table 1:** Trial inclusion and exclusion criteria

Inclusion criteria	Exclusion criteria
Homeless (living in temporary accommodation, no fixed abode or rough sleeping) [37] and Aged 18 years or over and One or more non-prescribed drug overdoses in past 6 months confirmed by self-report and witnessed overdose/ambulance call out/emergency department (ED) visit/naloxone use	Living in residential or community-based rehabilitation facility which has direct access to in-house medical and nursing care or Unable to give written informed consent

#### Setting

The study setting is Glasgow, Scotland (drug deaths account for 33.7 per 100,000 population and over half of all deaths in people experiencing homelessness (59%, 151 deaths)) [4].

Due to the risks associated with co-prescribing OST, diazepam and gabapentinoids together, specialist alcohol and drug recovery teams take responsibility for combination prescribing in people experiencing homelessness in Glasgow. Patients receiving these combinations tend to have their medicines dispensed daily, with consumption supervised in community pharmacies. For these reasons, overdose with prescribed medicines is less likely. The study therefore targeted people experiencing homelessness who had overdosed with non-prescribed (street) drugs.

In the UK, facilities for people experiencing homelessness who also have problem drug use, include residential rehabilitation units. These provide in-house short to medium term detoxification or stabilisation for people who have needs that cannot be met, although there are a shortage of rehabilitation beds. This level of respite care requires specialist addiction team input, and Glasgow is no different in this respect. Because of the level of specialist care needed to oversee stabilisation or detoxification, these units have qualified medical and nursing staff in-house. Clinical information relating to episodes of patient care in rehabilitation units include treatments is shared with the patient’s NHS primary and secondary care providers, to enable continuity of care after patients leave rehabilitation units. There are no barriers to information sharing with NHS practitioners including NHS employee PHOENIx Pharmacists. The PHOENIx team often refer patients into these residential rehabilitation units because their care needs cannot be met elsewhere.

In Glasgow, the care of problem drug or alcohol use in people experiencing homelessness with problem drug use is provided by specialist Alcohol and Drug Recovery Services (ADRS). Mental health services are provided by specialist homeless mental health teams, specialist community mental health teams or via ADRS. Mainstream General Practices, or the Specialist Homeless Health Service General Practitioner led service provide generalist care for physical and mental health needs, referring patients for specialist ADRS, mental health services and specialist hospital care as required. Community pharmacies dispense medicines but have no access to patients’ clinical information. Instead, community pharmacy staff retain their own records and share information about patients with ADRS staff as and when required. All health care (including medicines) is available free of charge in NHS Scotland. Social care and third sector charity services records are not routinely shared with other services. Prescribing for problem substance use is undertaken by ADRS; Glasgow also has Scotland’s first Heroin-Assisted Treatment Unit, with capacity for approximately 20 patients. Acute and long-term prescribing of most other medicines except antiretrovirals and some other specialised medication such as cancer chemotherapy is undertaken by General Practitioners and specialists in secondary care. Mental health teams (specifically psychiatrists) take responsibility for initiating antipsychotic medication. To have their health care needs met, patients with multimorbidity (two or more long-term conditions) [36, 38, 39] are therefore linked with at least two clinical services which are rarely collocated.

The PHOENIx intervention is described below, using the TIDieR checklist [40]. Full intervention details are described previously [36].

#### Background

Over 7500 (13%) of UK-based registered pharmacists have undergone additional subsequent training in therapeutics and completed a period of additional supervised clinical training, to gain an independent prescribing qualification enabling diagnosis and prescribing of common conditions. Independent prescribing pharmacists work in tandem with staff from Glasgow’s third sector homeless charities (the Simon Community Scotland and the Marie Trust) to offer the PHOENIx intervention. Previous qualitative work suggests benefit to patients [41], and a feasibility study describes the pharmacist assessing, treating, prescribing for acute and chronic health problems and referring for initiation of opioid substitution treatment (OST), while the homeless charity link worker addresses benefits, housing and social prescribing [33, 34].

#### Why

PHOENIx is a complex secondary prevention intervention. It is offered in addition to usual care, targeting people experiencing homelessness with recent overdose. It seeks to address overdose risk directly through conventional harm reduction (naloxone, same day access to ADRS for opioid substitution therapy) and offers assessment and immediate support for holistic health and social care needs, e.g. unmet mental, physical and social care needs [33, 34, 41]. This aims to improve access and continuity of care while reducing the number of services patients need to attend, and facilitating attendance at others, e.g. ADRS. PHOENIx aims to improve self-care and prevent deterioration in health through timely, immediate health and social care intervention on outreach.

#### Where

The PHOENIx team assertively outreach and deliver the intervention in various locations in Glasgow where people experiencing homelessness gather. This includes homeless congregate accommodation (large buildings with individual rooms, which do not have cooking facilities) housing multiple people experiencing homelessness.

#### How

The PHOENIx team always work in pairs. They access the patient’s existing NHS clinical records on a laptop with remote connection, while asking the patient to describe the health and social care problems that are important to them. These are recorded on paper forms and the patient’s clinical records. Through weekly conversations, PHOENIx build trusting therapeutic relationships with people experiencing homelessness, tackling problems in turn.

#### What

Working within the clinical governance framework provided by the patient’s General Practitioner and the local emergency department, the pharmacist leads on a full health assessment including measurements of weight, height, respiratory function and blood pressure, using routinely available NHS equipment. During consultations, the pharmacist, homeless worker and patient may decide to use standardised questionnaires as screening tools for common conditions: anxiety/depression (PHQ-4); modified Medical Research Council breathlessness score (mMRC) [42]; cardiovascular disease (ASSIGN); Malnutrition Universal Screening Tool (MUST); and alcohol screening (CAGE). Objective measures and subjective assessment scores help confirm diagnoses or severity of conditions. In some cases, depending on the patient’s clinical situation and priorities, these measures form an important part of the intervention when pharmacists chose to use them. They aid diagnoses and clinical decision-making in the clinical setting for pharmacists in the PHOENIx teams. Patients are routinely asked about common conditions including: hepatitis; HIV; dental problems; and injection site wounds; however, consultations follow the patient’s priorities and are personalised to their needs. The pharmacist listens, assesses and treats accordingly which may include a handwritten prescription (for any health condition), de-prescribing, and refers to a range of different health services as needed. The third sector worker manages the patient’s benefits claim support, offers social prescribing, advocacy, liaises with the patient’s existing support workers to optimise their accommodation and attends appointments with the patient if needed.

#### When and how much

PHOENIx aims to visit patients once weekly, and with consultations lasting an hour on average, the team follow patients wherever possible. Some patients require additional support and others require less, depending on the urgency, number of needs and patient preferences.

#### Who provided

PHOENIx staff are recruited based on their clinical independent prescribing (NHS Advanced Clinical Pharmacist) and housing (third sector worker) knowledge and skills, but also because of their street sense, empathy, active listening skills and non-judgemental attitude. These attributes were felt to be important to maximise the chances of immediately building rapport. People experiencing homelessness may have had difficulties forming and maintaining relationships because of past traumas [43], value receiving care directly rather than brokering [41, 44] and consider treatment for problem drug use to be effective when the care provider is compassionate and non-judgemental, taking time to understand the complexity of their lives [45].

Seven NHS employee pharmacists are available to deliver outreach visits, all working part time and three part time third sector outreach workers. Pairs attempt to retain contact with the same patients continuously.

#### Intervention fidelity

Assessed by the study lead, visiting PHOENIx teams on outreach every month, sitting in during consultations to check that the patient’s expressed needs were identified, and the team were supporting the patient with these needs and recording relevant information.

Full methods for the pilot RCT are described previously (https://doi.org/10.1186/ISRCTN10585019) [36]. Briefly, the main outcome is whether to progress to a subsequent definitive randomised controlled trial based on progression criteria: recruitment of ≥ 100 participants within 4 months; ≥ 60% patients remaining in the study at 6 -and 9-month follow-up; ≥ 60% participants in the PHOENIx group receiving the intervention; and ≥ 80% participants with data collected. Secondary outcomes include: rates and time to overdose; rates and time to hospitalisations; treatment uptake for physical health, mental health and problem drug use; health-related quality of life; and patient experience of treatment burden. Based on available guidance and data on recruitment and mortality from our feasibility study, we aimed to recruit at least 100 participants by inviting approximately 160, anticipating at least 64 with follow-up data to inform a sample size for a subsequent definitive RCT [33, 46].

#### Baseline assessments

**Fig. 1:**
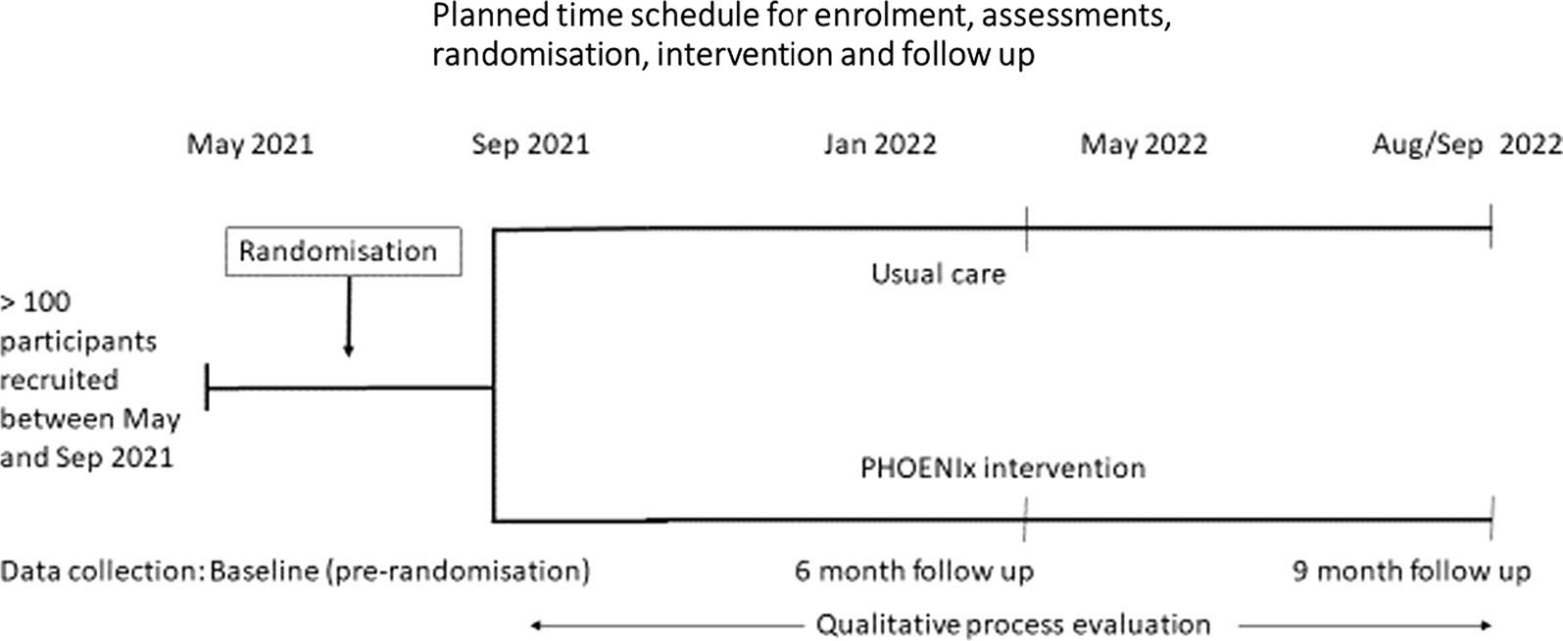
Trial schema

In-person baseline assessments and subsequent access by researchers to clinical and administrative records enabled gathering of comprehensive information (Additional file 2). Diagnoses data were collected through a combination of self-report and confirmed from medical records (Hospital, General Practice, or Alcohol and Drug Recovery Service clinical records). This is a necessary approach when collecting data in people experiencing homelessness because their lack of repeated engagement with primary medical care leads to low levels of General Practice registration. In turn, this means low levels of diagnoses recorded in General Practice clinical records and a requirement to access multiple clinical records to obtain diagnoses information. In addition, patient-reported information is important because people experiencing homelessness are itinerant, and may have registered with multiple General Practitioners, leading to missed information during transfers between care providers. Data items relate to the date of baseline data collection with the exception of questions about any overdoses, previous assaults and healthcare contacts which related to the period from 6 months prior to and including the date of baseline data collection, and blood results which were included if reported within a year of the date of baseline data collection. Trial schema is summarised in Fig. 1.

The research team cross-checked a 10% sample of data entries for accuracy and completeness. The Patient Experience with Treatment and Self-Management (PETS) questionnaire [47] assesses treatment burden in patients with chronic health conditions requiring self-management [47]. PETS had not been used previously in research with people experiencing homelessness; therefore, the research and clinical team worked with the developer (Dr David Eton) to adapt PETS version 2.0, to better suit the target group. The PETS including all modified, translated and adapted versions of it is protected by copyright, ©2020 Mayo Foundation for Medical Education and Research. All rights reserved. Permission to use the PETS can be sought from Dr Eton.

At 6- and 9-month follow-up, the research team will make repeated attempts to re-engage patients, as will the PHOENIx team during the intervention phase.

Descriptive outcomes will be conducted by independent statisticians after collection of 9-month follow-up data, using MINITAB statistical software (version 21) [48].

An embedded economic evaluation will examine the feasibility of determining the cost-effectiveness of the PHOENIx intervention in a subsequent definitive trial. The main analysis will consider a health and social care service perspective whereby unit costs are applied to each item of health (e.g. hospitalisation) and social care service use data. Unit costs will be taken from routine sources where possible including missed appointments [49–51]. The effectiveness of the intervention will be explored in terms of health state utilities (for a future cost utility analysis), as measured using the EQ-5D-5L to generate quality-adjusted life years (QALYs) to be used alongside the cost data to give an indicative picture of cost-effectiveness [52–54].

Qualitative components are embedded in this study, to enable an understanding of how participants respond to the intervention alongside an exploration of the contextual issues in which the RCT occurs [55]. This includes a process evaluation of the PHOENIx intervention and an exploration of participants’ perspectives of their drug use and overdoses, using normalisation process theory (NPT) to inform conceptualisation of the process evaluation data [56]. Coding will be conducted independently by NF and checked by the research team to reduce the risk of bias, ensure consistency and rigour. Data will be analysed using NVivo V.12 software [57].

### Baseline results

#### Recruitment

Visiting 20 different temporary accommodation venues across Glasgow city centre, researchers passed study information to staff [36]. Researchers (AMcP and JM) each had over 20 years of experience working with people experiencing homelessness and those with drug and/or mental health problems. Staff in homeless accommodations identified and approached patients they knew had overdosed in the preceding 6 months. Study information was received by people experiencing homelessness in low-threshold city centre venues, temporary accommodation or the street. Researchers offered a £10 shopping voucher to each participant on completion of baseline assessment.

One hundred and thirty eligible participants with at least one overdose in the past 6 months were offered recruitment across 20 different sites in Glasgow between 11 May and 1 September 2021. Two patients declined, leaving 128 who provided informed consent (Fig. 1).

Baseline interviews lasted approximately one hour. Following baseline interviews, which were conducted in person in the patient’s choice of venue, researchers accessed each patient’s multiple clinical and social care records: General Practitioner; hospital; ADRS/mental health; prescribing; social work; and third sector homelessness charity. Records were sought from NHS health board areas outside Greater Glasgow and Clyde if required. This enabled capture of complete data on diagnoses, laboratory tests, prescribing, healthcare contacts, housing and registration with services.

### Baseline demographic, physical and mental health characteristics (Table 2)

**Table 2:** Baseline demographic, physical and mental health characteristics (*N*% or mean (SD)/median (IQR)

Characteristic	PHOENIx participants (*n* = 128)
Age (years)	42.2 (8.4)
Sex (male)	91 (71.1%)
Body mass index (kg/m^2^)^a^	23.8 (5.1)
Underweight (< 18.5 kg/m^2^)	17 (14.7%)
Overweight/Obese (> 25 kg/m^2^)	45 (38.8%)
Ethnicity (White)	127 (99.2%)
Number of years experienced homeless^c^	23.5 (12–29.8)
*Temporary accommodation*	
Supported (hostel/low-cost hotel)	72 (56.2%)
Unsupported (hotel/bed and breakfast)	46 (35.9%)
Temporary Furnished Flat	3 (2.3%)
Rough Sleeping	5 (3.9%)
No fixed abode	2 (1.6%)
*GP registered (patient reported)*	
Mainstream GP	56 (43.8%)
Homeless Health Service GP	42 (32.8%)
Unknown	32 (25.2%)
Addictions Team registered^b^	113 (89.7%)
Homeless Addictions team	50 (39.1%)
Mainstream addictions team	63 (49.2%)
Mental Health Team registered^b^	43 (33.8%)
Homeless mental health team	13 (11.1%)
Mainstream mental health team	17 (14.5%)
Physical health conditions*	
Vascular	30 (23.4%)
Blood Borne Viruses	50 (39.0%)
Anaemia	3 (2.3%)
Skin	26 (20.3%)
Seizures	98 (76.5%)
Cardiovascular	15 (11.7%)
Chronic painful condition	20 (15.6%)
Fracture	13 (10.1%)
Alcohol related brain injury	1 (0.8%)
Respiratory	38 (29.6%)
Coronary heart disease	9 (7.0%)
Gastrointestinal (upper)	12 (9.4%)
Infection	11 (8.6%)
Epilepsy	13 (10.2%)
Alcohol related seizures	2 (1.6%)
Head/brain condition	2 (1.6%)
Neurological	11 (8.6%)
Chronic kidney disease	2 (1.6%)
Endocrine	16 (12.5%)
Genitourinary/pelvic	3 (2.3%)
Musculoskeletal	4 (3.2%)
Cachexia	2 (1.6%)
Rheumatic	3 (3.2%)
Wounds	42 (32.8%)
Dental condition	86 (67.2%)
Dentures	48 (37.5%)
Hearing condition	24 (18.8%)
Eye condition	72 (56.2%)
Head Injury	47 (36.7%)
Other	7 (5.5%)
Number of physical health conditions/patient	5.4 (2.5)
Patients with any physical health condition	124 (96.9%)
*Mental health conditions*	
Depression	85 (66.4%)
Anxiety	56 (43.7%)
Personality disorder	11 (8.6%)
Suicide attempt	73 (57.9%)
Mania/hypomania	1 (0.8%)
PTSD	25 (19.5%)
Complex trauma	10 (7.8%)
Childhood abuse/neglect	4 (3.1%)
Drug-induced psychosis	8 (6.2%)
Schizophrenia/psychosis	15 (11.7%)
Other mental health condition	26 (20.3%)
Number of mental health conditions/patient	2.2 (1.3)
Patients with any mental health condition	117 (91.4%)
*Psychological distress* ^e^	
(0–2 none; 3–5 mild; 6–8 moderate; 9–12 severe)	
PHQ-4	12 (8–12)
PHQ-4 ≥ 3	117 (92.1%)
Anxiety subscale	6 (4–6)
Anxiety score ≥ 3	108 (85.0%)
Depression subscale	6 (4–6)
Depression score ≥ 3	105 (82.7%)
*Any long-term health condition*	
0–1	0 (0%)
2–4	11 (8.6%)
5–8	59 (46.1%)
9–16	58 (45.3%)
Charlson comorbidity score^d^	2.8 (2.2)
Charlson 10-year survival percentage^d^	67.7 (34.9)
Assaulted (past 6 months)	58 (45.3%)
Feels unsafe	24 (18.8%)
No reported next of kin	39 (30.5%)

*Ever diagnosed (from self-report or medical records)

Missing data: ^a^*n* = 12. ^b^*n* = 1; ^c^*n* = 80

^d^Charlson comorbidity index calculator assesses the 10-year survival in mainstream housed patients with several comorbidities based on the CCI scoring system

^e^≥ 3 threshold for screening (data missing *n* = 1)

Data presented in Tables 2, 3, 4, 5 and 6 were obtained from self-report during in person assessments at baseline, and/or from case notes. Participants were on average 42.2 years old (SD 8.4), 91 (71.1%) male, 127 (99.2%) described their ethnicity as white, and had been experiencing homelessness for a median of 23.5 years (IQR 12–29.8). Participants lived in congregate temporary accommodation with half residing in city centre hostels or hotels, staffed by third sector homeless organisations. Others lived in low-cost congregate, emergency accommodation without any on-site support from dedicated homelessness workers, were sleeping rough, or had no fixed abode. Thirty-two (25%) participants were not registered with a General Practice. A total of 124 (96.9%) had at least one physical health problem, and 117 (91%) had at least one mental health problem. Forty-three (33.8%) were under the care of a mental health team. One hundred and thirteen (89.7%) were registered with specialist ADRS. Approximately one-third of participants reported being isolated with no friends or family, 24 (18.8%) felt unsafe, and almost half had been assaulted in the preceding 6 months.

Participants had a wide range of health conditions. The most common conditions were seizures, followed by dental problems, visual impairment, head injuries, wounds and respiratory conditions. Fifty (39%) of participants had blood borne viruses (Hepatitis C and/or HIV). The mean number of physical and mental health conditions per participant was 5.4 (SD 2.5) and 2.2 (SD 1.3), respectively. Eighty-five (66.4%) of participants had either self-reported and/or diagnosed depression, 56 (43.7%) had self-reported and/or diagnosed anxiety, 38(29.7%) had a history of suicide/self-harm and 25 (19.5%) had self-reported and/or diagnosed post-traumatic stress disorder. As psychological pain is a predictor of overdose risk [14], and levels of non-engagement with mental health services, we included the PHQ-4 questionnaire, which determines levels of psychological pain/distress in baseline assessments [58]. One hundred and twenty-seven (99.2%) participants completed the PHQ-4. Scores of three or over are diagnostic of clinically relevant psychological pain and/or distress, and 12 is the maximum score. The median score was 12 (IQR 8–12), signifying maximum levels of anxiety and depression.

### Overdose and problem drug use (Table 3)

Participants used a median of three different street drugs (IQR 2–4) in addition to OST, diazepam, and in one case, diamorphine from the Heroin-Assisted Treatment Unit. The mean (SD) number of overdoses in the past 6 months was 3.2 (3.2). A total of 81 (64%) participants were able to recall the drugs taken at the time of overdose; 65 (80%) identified street valium (benzodiazepine-type new psychoactive substance) [8] alone or with other substances as the main contributor. Half of participants described injecting drug use of whom half routinely accessed their femoral vein. Accessing either of the femoral veins constitutes risky injection practice because of the level of difficulty finding and accessing the vein and its proximity to the femoral artery. Multiple injection sites were common. Most participants (80 (68.4%) possessed naloxone. Table 3 also describes detailed patterns of use (frequency, dose and route) for each of the main street drugs. Most (112 (87.5%) participants took large amounts of street valium and 60% of participants used heroin and/or cocaine, mostly by injection. The majority (88%) smoked tobacco and 41% smoked cannabis. Forty-six (36%) reported daily alcohol consumption. The long-term nature of problem drug use was reflected by the ages at starting different drugs: on average, participants had their first cigarette and alcoholic drink aged 13 years, moving onto cannabis age 15 years, heroin age 20 years, cocaine age 30 years with street valium one and a half years later. Significant numbers (almost 20%) also bought and took street pregabalin or gabapentin.

**Table 3:** Baseline overdose and problem drug use (*N*% or mean (SD)/median (IQR)

Characteristic	PHOENIx participants *n* = 128
Number of overdoses in past 6 months^a^	3.2 (3.2)
1–2	70 (55.6%)
3–5	40 (31.7%)
6–10	14 (11.1%)
> 11	2 (1.6%)
Number of illicit drugs used concurrently^b^	3 (2–4)
Problem drugs used concurrently	
One	21 (16.4%)
Two	34 (26.6%)
Three	41 (32.0%)
Four	24 (18.8%)
Five	6 (4.7%)
Six	2 (1.6%)
*Main cause of overdose*^b1^	
Unable to recall	46 (36.0%)
‘Street Valium’	46 (56.8%)
‘Street Valium’ + other drugs	19 (23.4%)
Cocaine	5 (6.2%)
Heroin	6 (7.4%)
Suboxone	3 (3.7%)
Alcohol	2 (2.5%)
*Main route of drug administration*^b^	
Injection	65 (50.8%)
Injection Sites	
Groin	23 (35.4%)
Groin and leg	3 (4.6%)
Groin sinuses	6 (9.2%)
Arms and groin	1 (1.5%)
Arms	14 (21.5%)
Legs	8 (12%)
Hands	2 (3.1%)
All over	2 (3.1%)
Feet/neck	1 (1.5%)
Thigh	1 (1.5%)
Not Sure	1 (1.5%)
Number of injection sites^c^	
One to four	44 (34.4%)
Five to ten	15 (11.8%)
Too many to count	6 (4.7%)
Possesses naloxone^d^	80 (68.4%)
Knows how to use naloxone^d^	103 (88.0%)
*Heroin*	
Current^b^	77 (60.1%)
Frequency^e^	
Once or more daily/most days	36 (49.3%)
Every few days/weekly	9 (12.3%)
Every 2 weeks/monthly	10 (13.7%)
Rarely	18 (24.7%)
Dose^f^	
< 0.4 g (≤ £10)	16 (35.6%)
> 0.4 g but ≤ 2 g (£11–£50)	26 (57.8%)
> 2 g but up to 4 g (£51–£100)	3 (6.7%)
Route^g^	
Intravenous	36 (55.4%)
Snort	3 (4.6%)
Smoke	26 (40.0%)
Age started (years)	20.0 (16.3–26.0)
*Cocaine*	
Current	76 (59.4%)
Frequency^h^	
Once or more daily/most days	21 (28%)
Every few days/weekly	14 (18.7%)
Every 2 weeks/monthly	18 (24%)
Rarely	22 (33.0%)
Dose^i^	
< one bag (0.4 g; 2 lines); (≤ £10)	8 (18.6%)
> 1 bag—2 bags (£10—£20)	12 (27.9%)
> 2 bags—1 g (£21—£25)	4 (9.3%)
> 1 g (2.5 bags; > £25)	19 (44.2%)
Route^j^	
Intravenous	50 (73.5%)
Snort	10 (14.7%)
Smoke	8 (11.8%)
Age started (years)	30.0 (18.5–37.0)
*Street Valium*	
Current	112 (87.5%)
Frequency^j^	
Once or more daily/most days	71 (68.3%)
Every few days/weekly	15 (14.4%)
Every 2 weeks/monthly	9 (8.7%)
Rarely	9 (8.7%)
Dose^k^	
1–10 tablets	24 (26.5%)
11–25 tablets	35 (38.4%)
26–50 tablets	19 (20.9%)
51–100 tablets	10 (11.0%)
> 100 tablets	3 (3.3%)
Route^j^	
Oral	105 (100%)
Age started (years)^l^	31.5 (20.0–43.3)
*Spice*	
Current	8 (6.2%)
Only when in prison	8 (6.2%)
*Street gabapentinoids*	
Current	22 (17.2%)
*Cannabis*	
Current	53 (41.4%)
Frequency^n^	
Once or more daily/most days	23 (50.0%)
Every few days/weekly	7 (15.2%)
Every 2 weeks/monthly	2 (4.3%)
Rarely	16 (34.8%)
Age started (years)	15 (14–20)
*Tobacco*	
Current	113 (88.3%)
Cigarettes/roll-ups/day	15.7 (15.2)
Age started (years)	13.0 (5–15)
*Alcohol*	
Daily drinking	46 (36.0%)
Units/week (recommended 4u maximum)	200.7 (151.1)
Age of first drink (years)^m^	13 (11.5–15.0)
Previous withdrawals/DTs	38 (82.6%)
Previous detox/rehab for alcohol	30 (65.2%)

^a^Self-reported; ^a^data missing *n* = 2. ^b^In past 6 months. ^b1^Data missing *n* = 1. ^c^From 65 respondents reporting injecting drug use. ^d^Data missing *n* = 11. ^e^*n* = 4; ^f^*n* = 32; ^g^*n* = 12; ^h^*n* = 1; ^i^*n* = 33; ^j^*n* = 8; ^k^*n* = 21; ^l^*n* = 38; ^j1^Street Valium + hash; ^m^Data from *n* = 105; missing *n* = 7

### Prescribed medicines (Table 4)

More participants took daily prescribed OST (115 (89.8%) than smoked or injected heroin (77 (60.1%). In contrast, fewer people (13 (10.2%) received prescribed diazepam, than reported problem street valium use (112 (87.5%). None of the participants reported receiving counselling or other psychological behavioural therapies. Ninety-one per cent of participants had at least one (self-reported and/or confirmed by case notes) mental health problem; however, only half (67 (52.3%) were receiving any mental health treatment, most commonly antidepressants followed by anxiolytics (including diazepam) and antipsychotics.

Almost all (97%) of participants had multiple treatable current physical health problems (Table 2), but only 65 (50.8%; Table 4) were receiving any treatment, the most prevalent being analgesics, medicines to treat nutritional deficiencies or anaemias, respiratory problems, upper gastrointestinal problems and antiretrovirals for blood-borne viruses. The first and second COVID-19 vaccines had been administered to most of the Scottish population in the period May–September 2021 [59]; however, only 23 (18%) of participants reported receiving both, and 28 (21.9%) reported receiving their first dose on﻿ly.

**Table 4:** Prescribed medicines (*N*% or mean (SD)/median (IQR)*

Medicine	PHOENIx participants
	*n* = 128
Opiate substitution treatment	115 (89.8%)
Methadone	95 (74.2%)
Daily dose (mg)	86.9 (29.4)
Buprenorphine oral/sublingual/with naloxone	17 (13.3%)
Daily dose (mg)^a^	16 (10–19.5)
Buprenorphine injection	2 (1.6%)
Weekly dose (mg)	96 (64–128)
Diamorphine injection	1 (0.8%)
Daily dose (mg)	300 (–)
Diazepam treatment	13 (10.2%)
Daily dose (mg)	21.3 (8.9)
Number of medicines for problem drug use	1 (0–1)
Any medicine for problem drug use	115 (89.8%)
*Medicines for mental health problem*	
Any mental health medicine	67 (52.3%)
Number of medicines for mental health problem	1 (0–1)
Type of medicine for mental health problem	
Antipsychotic	19 (14.8%)
Antidepressant	53 (41.4%)
Anxiolytic^b^	23 (17.9%)
*Medicines for physical health problem*	
Any medicine for physical health problem	65 (50.8%)
Number of medicines for physical health problem	1 (0–2)
Type of medicine for physical health problem	
Nutrition and anaemia	21 (16.4%)
Analgesic	32 (25.0%)
Topical for skin condition	2 (1.6%)
Antiepileptic	7 (5.5%)
Nocturnal leg cramps	1 (0.8%)
Upper gastrointestinal	11 (8.6%)
Laxative	1 (0.8%)
Respiratory	14 (10.9%)
Diabetes	4 (3.1%)
Antiretroviral	10 (7.8%)
Antibacterial/antifungal	5 (3.9%)
Antiplatelet	2 (1.6%)
Diuretic	2 (1.6%)
Statin	3 (2.3%)
Sex hormone	1 (0.8%)
Antihypertensive	5 (3.9%)
Hormone Replacement Therapy	2 (1.6%)
Drug for movement disorder	1 (0.8%)
COVID-19 vaccine^c^	
None	23 (18%)
Declined to answer question	54 (42.2%)
1st dose only	28 (21.9%)
1st and 2nd doses	23 (18.0%)

*Current

Data missing ^a^*n* = 1; ^b^Diazepam. ^c^Booster unavailable at time of recruitment

### Baseline function, quality of life and objective health measures (Table 5)

Frailty is a syndrome of vulnerability conferring an increased risk for falls, disability, hospitalisation and mortality [60]. Frailty was examined because of our previous finding that people experiencing homelessness in Glasgow and Edinburgh, despite being 43 years old on average, had high levels of multimorbidity comparable to people aged 85 years in mainstream society [22]. We used an adapted Fried’s frailty phenotype [60] (Table 5) which included five measures assessed through standard questions (unintentional weight loss; self-reported exhaustion; low physical activity; and slow walking speed) and weakness (through a hand dynamometer). Participants with three or more scores above the relevant threshold for each measure are considered frail, and those with one or two criteria are pre-frail. Of the 71 participants with all five measures available, most (50/71 (70.4%) were frail and all but one of the remainder were pre-frail.

Table 5 describes EQ-5D-5L data, which enabled participants to rate their health under five domains: mobility; self-care; usual activities; pain/discomfort; and anxiety/depression. Each domain had five possible answers ranging from the participant being unable to walk about, wash/dress or self-care, having extreme pain/discomfort/anxiety or depression (all scored as 5), to having no problem with any of the domains (scoring 1). A separate visual analogue scale ranging from 0 (worst possible health) to 100 (best possible health) enabled participants to rate their health. Information was available for 125 participants (98%) although two of these participants only completed the visual analogue scale section and so indexed data (cross-walked to the EQ-5D-3L value set for the UK) [53] were available for 123 participants. Overall, reported domain scores were highest (indicating poorer quality of life) at 4 (IQR 3–5) for the “depression/anxiety” domain. The “mobility” and “activities of daily living” and the “pain/discomfort” domains were rated as 3 (2–4).

Table 5 describes meals received by participants. Single rooms in temporary accommodation had no cooking facilities and given the level of destitution associated with being homeless, participants relied on food handouts from their accommodation or soup kitchens. Most participants (except those with no fixed abode or in temporary furnished flats, where they have no immediate access to ready meals) had in-house, ultra-processed ready to eat or heat meals, soft drinks, crisps, packaged snacks, commercial bread, cakes and biscuits (particularly shortbread), sweetened breakfast “cereals”, sugared milk-based and “fruit” drinks. Temporary accommodation had one communal microwave in the reception area, for large numbers of residents. Approximately 60% of participants had fewer than three meals/day and 20 (16%) had no daily meals, living on snacks. The majority were either underweight (15%) or overweight/obese (40%).

### Objective health measures (Table 5)

Objective measures of respiratory status at the time of baseline interviews included an assessment of the functional impact of breathlessness using the modified Medical Research Council breathlessness scale [42]. The scale ranged from 0 (breathless only on hard exercise) to 4 (too breathless to leave accommodation), and most participants scored 2 (on level ground, participants walk slower than people of the same age because of breathlessness, or have to stop for breath when walking at their own pace on the level). Twenty-two (17%) had oxygen saturation measurements less than 95% at rest. Mean systolic blood pressure was 115.8 (SD18.5). Biochemical values were collected from medical records, expressed as the proportion out with NHS Greater Glasgow and Clyde laboratory reference ranges and mean (SD). In most cases, samples were taken from visiting ED or during a hospital inpatient episode of care rather than for screening purposes. 

**Table 5:** Baseline functional, quality of life (*N*%) or mean (SD)/median (IQR)

	PHOENIx participants (*n* = 128)
*Frailty criteria* ^h^	
Weight loss	67 (65%)
Exhaustion	92 (77.3%)
Low activity	88 (69.8%)
Slowness	75 (66.4%)
Weakness	28 (25.2%)
Positive for frailty phenotype	50 (70.4%)
Pre-frail	20 (28.2%)
Quality of life (EQ5D5L)^b^	
Mobility	3 (1–4)
(1 = no problem; 2 = slight; 3 = moderate; 4 = severe; 5 = unable to mobilise)	
Self-care	2 (1–3)
(1 = no problem; 2 = slight; 3 = moderate; 4 = severe; 5 = unable to self-care)	
Usual activities	3 (1–4)
(1 = no problem; 2 = slight; 3 = moderate; 4 = severe; 5 = unable to do usual activities)	
Pain/discomfort	3 (2–4)
(1 = no problem; 2 = slight; 3 = moderate; 4 = severe; 5 = extreme pain/discomfort)	
Anxiety/depression	4 (3–5)
(1 = no problem; 2 = slight; 3 = moderate; 4 = severe; 5 = extreme)	
Overall health number—Visual Analogue Scale (VAS)^c^	34.4 (23.9)
(0 = worst health imaginable; 100 = best health imaginable)	
Index Score—crosswalk method to UK Value Set	0.2 (0.3)
(− 0.5 = lowest score on all five domains; 1 = highest score on all five domains)	
Meals^c1^	
Breakfast, lunch and dinner	35 (28.5%)
One meal only per day	34 (27.6%)
Two meals only per day	34 (27.6%)
No daily meals	20 (16.3%)
Modified Medical Research Council breathlessness scale^d1^	2 (1–3)^d^
Oxygen saturation (%)^g^	96.4% (2.3%)
Peak expiratory flow rate (% predicted, l/min)^i^	70.6 (21.5)
Systolic blood pressure (mmHg)^e^	115.8 (18.5)
Heart rate (beats per minute)^f^	78.3 (14.0)
Sodium (133–146 mmol/l)^k^	103 (95.4%)
Mean (SD)	138.5 (3.2)
Potassium (3.5–5.3 mmol/l)^l^	104 (95.4)
Mean (SD)	4.2 (0.5)
Creatinine (40–130umol/l)^m^	108 (99.1%)
Mean (SD)	68.4 (15.1)
Estimated GFR (% > 60 ml/min)^m^	106 (97.2%)
Alanine aminotransferase (% < 50 U/L)^n^	86 (80.4%)
Mean (SD)	30.9 (28.6)
Asparate aminotransferase (% < 40 U/L)^0^	80 (75.5%)
Mean (SD)	40.2 (40.7)
Alkaline phosphatase (< 130 U/L)^0^	89 (84.0%)
Mean (SD)	103.9 (56.1)
Albumin (> 35 g/l)^m^	63 (57.8%)
Mean (SD)	36.5 (5.6)
Calcium (adj 2.2–2.6)^t^	77 (89.5%)
Mean (SD)	2.3 (0.1)
Magnesium (> 0.7 mmol/l)^p^	52 (81.2%)
Mean (SD)	0.8 (0.2)
C-reactive protein (< 10 mg/l)^q^	53 (51.5%)
Mean (SD)	19.4 (28.7)
B_12_ (200–883 ng/l)^r^	20 (90.9%)
Mean (SD)	508.3 (217.4)
Folate (serum: 3.1–20.0 ng/ml)^s^	11 (47.6%)
Mean (SD)	4.3 (2.4)
Red cell count (4.5–6.5)^u^	33 (31.1%)
Mean (SD)	2.3 (0.1)
Platelets (150–410)^u^	96 (90.6%)
Mean (SD)	258 (85.6)

Data missing ^b^*n* = 5; ^c^n = 3. ^c1^n = 5. d, e, f, g, h and i collected at interview. k through to s: collected from clinical records most recent in past year. ^d1^ Options 0 (breathless only on hard exercise-1-2-3-4 (too breathless to leave accommodation). ^h^Frieds frailty phenotype (adapted): ≥ 3 criteria = positive; 1 or 2 criteria = intermediate or pre-frail. ^d^“Walk slower than other people of same age or stop for breath when walking at own pace” data missing *n* = 13; Data missing ^e^*n* = 5; ^f^*n* = 4; ^g^*n* = 3; ^h^*n* = 57; ^I^missing data *n* = 12; Data missing ^k^*n* = 20; ^l, m^*n* = 19; ^n^*n* = 21; ^o^*n* = 22; ^p^*n* = 64; ^q^*n* = 25; ^r^*n* = 106; ^s^*n* = 108; ^t^*n* = 42; ^u^*n* = 22

### Healthcare utilisation in preceding 6 months (Table 6)

One-third of participants had been in contact with a General Practitioner and fewer had received care from a General Practice-based nurse or other healthcare professional. In contrast, two-thirds of participants (80 (62.5%) had received at least one consultation with a nurse from the ADRS, and similar numbers had received care from social care staff. Between 15 and 16% had received input from an addictions doctor or non-medical (ADRS) prescriber, respectively. One quarter of participants had received care from a mental health nurse during the previous 6 months, and fewer than one in 20 had received input from a psychiatrist.

**Table 6:** Healthcare contacts in past 6 months (*N*% or mean (SD)/median

Characteristic	PHOENIx participants *n* = 128
*Primary care*	
General Practice (specialist homeless or mainstream)	
Contacts/patient^a^	0 (0–1)
Patients with GP contact	40 (31.2%)
GP-based physical health nurse consultations/patient	1 (1–2)
Patients with GP-based physical health nurse contacts	20 (15.6%)
Other primary healthcare staff consultations/patient^b^	0 (0–0)
Patients with other primary healthcare contacts^b^	14 (10.9%)
Alcohol and Drug Recovery Service	
Nurse contacts/patient	1 (0–4)
Patients with any ADRS nurse contacts	80 (62.5%)
Pharmacist contacts/patient	0 (0–0)
Patients with any addictions pharmacist contacts	20 (15.6%)
Medic contacts/patient	0 (0–0)
Patients with any addictions medic contacts	19 (14.8%)
Mental health (specialist homeless or mainstream)	
Mental health nurse contacts/patient	0 (0–1)
Patients with any mental health nurse contacts	33 (25.8%)
Consultant psychiatrist contacts/patient	0 (0–0)
Patients with any consultant psychiatrist contacts	5 (3.9%)
Social care	
Social care staff consultations/patient	1 (0–4)
Patients with any social care contacts	79 (61.7%)
*Hospital*	
Mental health	
Mental health hospitalisations/patient	0 (0–0)
Patients with any mental health hospitalisation	8 (6.2%)
Duration of mental health hospitalisations (days)	11.5 (5.0–22.0)
General hospital	
Emergency department (ED) contacts/patient	3 (1–5)
Patients with any ED contacts	105 (82.0%)
Hospitalisations/patient	1 (0–2)
Patients with any general hospitalisations	89 (69.5%)
Duration of general hospitalisations (days)	2 (1–4)
Outpatient clinic attendance/patient	1 (0–2)
Patients with any outpatient attendances	66 (51.6%)
Outpatient clinic appointments not attended	0 (0–3)
Patients with ≥ 1 non-attendance at outpatient clinic	51 (39.8%)
Rehabilitation for drug use (residential)	
Residential rehabilitation stays/patient	0 (0–0)
Patients with any residential rehab stays	5 (3.9%)
Duration of rehab (days)	21.5 (11.5–23.8)

^a^Homeless Health service GP/mainstream GP

^b^Includes Occupational Therapist, Dietician, Podiatrist, sexual health nurse and others, excludes addiction and mental health team

Participants had a median of three ED visits in the past 6 months, 82% having visited ED at least once. Seventy per cent had spent time in the local general hospital although unlike mental health admissions where the median length of stay was 11.5 (5–22) days, the median length of stay in the general hospital was 2 (1–4) days. Half of participants had attended, and 40% had not managed to attend at least one scheduled outpatient appointment.

## Discussion

Despite having a reputation for being hard to reach, 128 from 130 participants were engaged, provided consent and detailed baseline information during lengthy in person assessments in one of 20 different venues.

The median duration of homelessness was 23.5 years, which is approximately half the life expectancy of a person experiencing homelessness in Scotland [3, 11, 61]. Participants had pervasive, high-risk polydrug use, using a median of three different street drugs in addition to prescribed OST and in some cases, prescribed diazepam. Participants had a mean of three non-fatal overdoses in the previous 6 months. Participants were frail and had a greater number of health conditions than people double their age in mainstream society [62], conferring a high level of susceptibility to, and impact from overdose. Most participants were known to and receiving OST from ADRS. However, participants continued to overdose, in most cases, with street valium, for which there is no strong evidence base for treatment [8, 24] although one in ten were prescribed maintenance diazepam.

Heroin-assisted treatment of problem opiate use has some evidence of reduced street heroin use; however, the impact on overdose remains uncertain [63]. Patients with active significant medical or psychiatric conditions were excluded from the most recent, definitive RCT of heroin-assisted treatment which included 127 participants [63]. One hundred and twenty-four (96.9%) participants had active significant physical health problems, and 117 (91.4%) had psychiatric conditions: the majority would have been excluded from the most recent trial of heroin-assisted treatment [63]. This makes it difficult to generalise the utility of prescribed heroin to our cohort.

The effectiveness of current care for problem drug use in people experiencing homelessness could be assessed by measures such as the number of drug-related deaths or overdoses or the number of participants presenting to emergency department with drug-related problems. Given one of the main outcomes from treatments such as OST and diazepam is to reduce harm and prevent overdose and deaths, the effectiveness of current care appears conditional on the effectiveness of these treatments for poly problem drug use. That participants repeatedly overdosed despite receiving OST and in some cases, diazepam, highlights an evidence and practice gap in the care of participants in this study, in terms of their problem drug use. In terms of established interventions, OST is proven to reduce all cause and overdose mortality in people dependent on street opioids [21]. However, it remains uncertain whether people currently experiencing homelessness with polydrug use including opioids, accrue these benefits because of exclusions from previous RCTs [21]. The extent of ongoing polydrug use including street heroin (60% of participants, 49% of whom use at least 0.4 g once daily (Table 3) suggests more studies are required to examine whether OST at optimal dose (mean 87 mg) (Table 4) prevents illicit drug use and overdose in our cohort. People experiencing homelessness were largely absent from trials of OST, and participants taking three different street drugs were also absent, meaning there is a lack of evidence of benefit in the type of patients within our cohort [21]. In addition, now that the characteristics of people experiencing homelessness with recent overdose are known (Tables 2, 3, 4, 5, 6), a comparison with existing literature shows there are no established interventions known to reduce overdoses or emergency department visits in this type of cohort (Additional file 1).

Difficulties associated with recruitment of people experiencing homelessness in trials may have previously limited collection of information about characteristics [64, 65]. Our recruitment rate (128 participants from 130 potential participants) was higher than expected. This may have been due to close collaboration between researchers and third sector homeless charity workers, who had established relationships with eligible participants. Outreaching to participants in their own spaces enabled engagement. Participants were offered a shopping voucher on completion of baseline interviews. This and the non-judgemental, empathetic approach by the researchers may have contributed to high recruitment rates. A parallel process evaluation is underway and will capture information on barriers and facilitators to recruitment from the perspectives of participants and staff.

“Housing First” offers permanent, self-contained housing for people experiencing homelessness, alongside wrap-around health and social care support. It is an evidenced approach to ending homelessness for people with complex needs including mental health problems [16, 17, 66]. In this sample, no participants were being considered for Housing First accommodation, at baseline, despite being eligible for Housing First. It is out with the scope of this pilot RCT to fully explore why this was the case.

Participants had an average of two mental health problems. Medicines and other approaches have a role in the treatment of people with complex trauma and other mental health problems. Sixty-seven (50%) were in receipt of medicines for mental health problems, but none of the 128 participants were receiving any other form of specialist mental health input. While this is in keeping with previous work [67], restrictions due to COVID-19 may have contributed to participants experiencing difficulties accessing mental health services, particularly when digital options were not available to those in temporary accommodation. Participants had more physical health problems than mental health problems. Only half of those with physical or mental health problems were receiving any treatment, a finding consistent with the rule of halves [68].

Residential treatment services for drug use are scarce across Scotland [69]. Our findings confirmed less than one in 20 participants had received residential treatment in the previous 6 months. This may represent low uptake, but comparisons are not possible because previous studies of people experiencing homelessness have not recruited a comparable sample in terms of range and chronicity of drug use with complex physical and mental health problems (Additional file 1) [15].

Inequities in the prescribing of diazepam may be due to clinical decision making in the light of uncertain evidence of benefit particularly in a high-risk cohort using multiple drugs who are frail. Given the high prevalence and importance of problem street diazepam use in our participants, and the congregate living conditions which bring participants into close proximity, it is likely that participants are aware of each other’s drug habits and treatments. Prescribed diazepam inequalities are unlikely to be lost on participants who already have a heightened sense of discrimination and stigmatisation and live together. Other unexplained inequities shown by these data include: variable uptake of COVID-19 vaccination; irregular registration with General Practitioners; and low levels of registration with mental health services.

Health-related quality of life is regarded to be the most relevant outcome for people experiencing homelessness; health outcomes are significantly associated with quality-of-life scores [70, 71]. Participants’ current situation, plus the cumulative long-term impact of severe and multiple disadvantage, was manifest in quality-of-life findings which were rated in the bottom third of the EQ-5D Visual Analogue Scale. Patient responses to EQ5D5L scores are matched to a general population sample that has previously rated every possible response combination to the questionnaire’s five domains, to estimate how much the population values being in (or avoiding) that particular health state [54]. These population values range from 1 (full health) to a minimum of − 0.224, beyond the zero score for death. This accounts for the possibility that there are some health states the public would prefer to avoid so much that they would rather be dead. Matching PHOENIx participants to these scores showed one-third of respondents at baseline were in health states considered “worse than death”. Quality of life offers a possible primary outcome measure in the future randomised controlled trials of people experiencing homelessness.

Our findings demonstrate extensive unmet health and social care needs of people experiencing homelessness post overdose. These needs are unlikely to be met by continuation of care as usual. Innovative models of care and new interventions are necessary to address the status quo, accompanied by robust, pragmatic research including qualitative research to understand the complexity and barriers and facilitators to real world implementation [1, 72–74]. The PHOENIx intervention and RCT offers a novel, generalist approach instead of the current problem drug use oriented approach which characterises usual care. PHOENIx acknowledges patients’ priorities, and their multiple and competing relational, social care and health problems including maximum levels of frailty, anxiety and depression, which contribute to overdose risk [14]. People experiencing homelessness are known to have more difficulty using fragmented care systems, as compared with people without multiple health needs [62, 71, 75]. The existing evidence base for reducing drug-related deaths does not favour the current approach of tackling single morbidities, e.g. problem drug use, in isolation [1, 30, 76]. People experiencing homelessness do not favour the current approach either. [30, 76]. Together, the range and complexity of life threatening problems and under treatment characterising study participants makes a case for testing a transformational approach to offering and providing comprehensive, continuous and co-ordinated health and social care.

The competing needs of finding safety, managing the impact of an accumulated treatment burden and self-medicating for anxiety and substance dependence may have diverted attention away from health seeking behaviour until problems became overwhelming and required ED attendance [77]. The alliance–outcome relationship is one of the strongest predictors of treatment success [78]. We hypothesise that supportive relationships built through outreach may prevent or delay emergency department attendance if the skills and knowledge of those delivering outreach are sufficient to deal with most of the patient’s problems. Supportive relationships in conjunction with practical, immediate help with a range of health and social care problems are core features of the PHOENIx intervention [41].

Limitations to generalisability include most of the participants identifying as Caucasian, and recruitment from one Scottish city albeit across 20 different venues. Screening for other specific conditions, e.g. atrial fibrillation through electrocardiography or blood samples for nutritional deficiencies, did not form part of baseline assessments which limits our understanding of these and other important needs. Worldwide, proportions of people experiencing homelessness using multiple street drugs and overdosing are unclear, making generalisations based on these data difficult. There were 26,166 homelessness applications across Scotland in 2021/2022 [79]. The number of people experiencing homelessness with problem drug use in Glasgow is in the region of 3500 [22]; however, numbers overdosing remain uncertain, making it difficult to know whether findings from 128 participants are generalizable. Characteristics of people dying drug-related deaths show the mean age has increased from 35 years in 2009, to 42 years in 2018, and the most commonly implicated substances were street benzodiazepines, methadone and heroin/morphine [4, 80, 81]. Our sample demographic is a close match to the characteristics of those experiencing homelessness and dying from drug-related causes in Scotland as a whole [3]. The number of participants in our pilot RCT is comparable to the numbers recruited in previous (definitive) studies (Additional file 1) [15].

## Conclusion

People experiencing homelessness with recent overdose can be recruited, and their characteristics can be described through comprehensive baseline data collected in the context of a pilot RCT.

Complex drug use and frequent overdose combined with multiple unmet health needs. This suggests the current focus on stabilising street drug use and reducing harm from drugs without attention to wider health and social care needs including unstable housing, are failing to protect against non-fatal and by inference, fatal overdose. Current models of care in Glasgow and worldwide (Additional file 1) [15] tend to focus on single conditions, an approach that does not seem sensible when multimorbidity, re-traumatising living conditions [82] and frailty are the norms. This signals an urgent need for broadening the scope of support offered on outreach, to include a health and social care partnership, to address wider determinants of non-fatal and fatal overdoses.

If retention and intervention delivery targets are achieved, together with a signal of improvement in outcomes such as overdoses or quality of life, funding will be sought for a definitive RCT of the PHOENIx intervention.

## Supplementary Information

**Additional file 1.** RCTs of interventions to improve health outcomes.

**Additional file 2.** Baseline data collection form.

## Data Availability

All data used during the study are available on reasonable request from the corresponding author.
