# Supplementary material for: Baseline characteristics of people experiencing homelessness with a recent drug overdose in the PHOENIx pilot randomised controlled trial
Source: Harm Reduct J. 2023 Apr 4;20:46. doi: 10.1186/s12954-023-00771-4 (PMC10071267; doi:10.1186/s12954-023-00771-4)
Supplement: Supplementary file 1 — Additional file 1. RCTs of interventions to improve health outcomes. [file 12954_2023_771_MOESM1_ESM.docx]

**Supplementary Appendix 1.**

**Figure 1 Search strategy Dec 2017 – January 2022**

Records identified through database searching-using updated search (Dec 1^st^ 2017-January 31^st^ 2022):

MEDLINE n = 37; Embase n = 77; PsychINFO n = 50; CENTRAL n = 132

Full articles excluded n = 249:

Not relevant n = 81

Not homeless n = 51

Not adults n = 10

Not RCT n = 88

Results not available (ongoing trial) n = 5

Drug misuse not included in baseline data n = 5

Papers excluded because described same study n = 9

Studies identified as relevant n = 11:

Psychinfo n = 0

Medline n = 6

EMBASE n = 2

Central n = 3

Full text articles assessed for eligibility n=260

Records after duplicates removed n = 260

**Table 1. Randomised controlled intervention trials aiming to improve health outcomes in homelessness post 2017 (N% or mean (SD)/ median (IQR)**

|  | **Lowrie et al**  **N=128** | **Chum et al**  **N=575** | **Aubry et al**  **N=201** | **Bring et al**  **N=96** | **Towe et al**  **N=236** | **Collins et al**  **N=169** | **Nyamathi et al N=130** | **Salem et al N=703** | **Malte et al**  **N=181** | **Tinland et al N=703** | **Nyamathi et al**  **N=600** | **Nyamathi et al**  **N=422** |
| --- | --- | --- | --- | --- | --- | --- | --- | --- | --- | --- | --- | --- |
| Type of homelessness  Single room/cheap hotel | 118(92%) | - | - | - | **-** | **-** | **-** | **-** | **-** | - | - | - |
| Temporary flat  No fixed abode | 3(2%)  2(2%) | - | **-**  **-** | -  - | **-**  **-** | **-**  **-** | **-**  **-** | **-**  **-** | **-**  **-** | - | -  - | - |
| Rough sleeping  Shelter  Additional inclusion criteria | 5(4%)  -  Recent drug overdose | -  -  Mental Health disorder | -  -  Mental Health disorder | -  -  Admitted to hospital | -  -  HIV +ve | -  -  Alcohol use | -  -  Current prisoner | 37.1%  46.9%  >40 years | -  Army Veteran | -  -  Mental Health | -  -  Ex-offenders | 61.7%  13.98%  Gay/bisexual men |
| Age (years) | 42(8.4) | 25-49y (66.5%) | 18-40y (49.2%) | 48 | 46.3 | 47.86 | 38.9 | 54.78 | 50.6 | 38.8 | 40 | 34.42 |
| Sex (% male) | 91(71%) | 67.1% | 64.6% | 91% | 73% | 76% | 0% | 0% | 97.8% | 82.5% | 100% | 100% |
| Recruitment period | May-Sep 2021 | Oct’09-Jul’11 | April’11-Feb’13 | April’14-March’16 | April ’12-13 | Oct 15-Feb17 | Feb ‘15-Nov ‘16 | July-Nov ‘14 | Oct ’11-Nov ‘15 | Aug ’11-Nov ‘14 | Feb’10-Jan’13 | Jul ’09-Feb’15 |
| Location | Scotland | Canada | Canada | Denmark | USA | USA | USA | USA | USA | France | USA | USA |
| Follow up duration | 9 months | 24 months | 24 months | 12 months | 12 months | 3 months | 6 months | 1 month | 24 months | 24 months | 12 months | 8 months |
| N(%) retained (Intervention (Ix)/Control) |  | 87%Ix/74%  control | 90%Ix/77.2% Control | 96%Ix/96%control | 94.1%Ix/97%control | 76.7% | 90.1%Ix/87.7%Control | 86.7%Ix/88.2% Control | 100%Ix/100%control | 78%Ix/63%Control | 89.7% | 86.8%Ix/88.6%Control |
| Homelessness (years) | 23.5(12-29.8) | 77.7% >1yr | 66.1%>1yr | - | - | - | - | - | 33.5%>1yr | 6yrs (median) | - | - |
| Health problems |  |  |  |  |  |  |  |  |  |  |  |  |
| Mental health^a^ | 117(91%) | 100% | 100% | 48% | 58% | - | 44.6%^b^ | 77.4% ^b^ | 80% | 55.4% ^b^ | - | 12.87% ^b^ |
| Physical health^a^ | 124(97%) | - | 89% |  |  |  |  | 87.5% | - |  | 33.67%^c^ | 25.66% ^c^ |
| Drug use | (99%;39% IV) | 21.6%^d^ | 53.2% | 87% | 37%(IV) | 78%(>1 drug) | 68.5%^e^ | 28.1% ^e^ | 36%^e^ | 46.3% | 60%^f^ | 34.14%(IV) |
| Alcohol use | 46(36%) | - | 31.3% (addiction) | 70% | 64% | 82% | 41.5%(last 6mo) | 53.1%(last 6mo) | 64.1% | 39.3% | 63.98% (binge drink) |  |
| Intervention | Weekly pharmacist/homeless worker outreach for 6-9 months | Housing first | Housing first | Medical Respite | Rapid rehousing | Alcohol harm reduction | Dialectical behavioural therapy vs Health Promotion | Frailty intervention vs Health Promotion | Addiction/housing case management | Housing first | Nurse delivered intervention | Nurse education |
| Primary outcome  Economic evaluation  Qualitative process evaluation | Progression to definitive RCT, overdoses, HRQoL  Yes  Yes | Improvement of CV risk factors  No  No | Housing stability  No  No | Health care costs  Yes  No | Housing stability  No  No | QoL, alcohol reduction  No  No | Drug and alcohol abstinence  No  No | Frailty scores  No  No | Housing stability  No  No | No. of hospital visits  Yes  No | Reducing re-arrests  No  No | HBV vaccine status  No  No |

^α^ Antiretroviral therapy^; β^ Intervention; **^∞^** 23% homeless participants; ^Ω^ self report; ^µ^ for primary outcome; ^§^ record linkage; ^a^ at least one condition ^b^Depression ^c^self reported fair/poor health ^d^ Used crack or cocaine last 30d ^e^any drug ^f^cocaine
