# Supplementary material for: Baseline characteristics of people experiencing homelessness with a recent drug overdose in the PHOENIx pilot randomised controlled trial
Source: Harm Reduct J. 2023 Apr 4;20:46. doi: 10.1186/s12954-023-00771-4 (PMC10071267; doi:10.1186/s12954-023-00771-4)
Supplement: Supplementary file 2 — Additional file 2. Baseline data collection form. [file 12954_2023_771_MOESM2_ESM.docx]

**Baseline data collection form**

| Baseline Date: | _____________________________ | Location: | _____________________________ | |
| --- | --- | --- | --- | --- |
| Completed by: | _____________________________ | How identified? | _____________________________ | |
| Advise voucher at end of interview  (£10 baseline interview, £10 subsequent interviews –6 & 9 months post baseline) | | | Yes ❑ | No ❑ |

**COVID Screen**

| Temp: | | Have you had the Covid vaccine? | | 0 ❑ / 1^st^ ❑ / 2^nd^ ❑ | |
| --- | --- | --- | --- | --- | --- |
| O_2_ Sats: | | Pulse: | | RR: | |
| New, dry cough >2 days? | Yes ❑ / No ❑ | New Breathlessness? | Yes ❑ / No ❑ | Anosmia? | Yes ❑ / No ❑ |

| ***Temp >38 / RR >25 breaths per min / HR >110 🡪 arbitrary cut off for severe exacerbation/sepsis*** |
| --- |

**1 DEMOGRAPHICS**

| Patient Name: | _____________________________ | Date of Birth: | _____________________________ |
| --- | --- | --- | --- |
| Sex: | Male ❑ / Female ❑ / Other ❑ | Age of Patient at interview date: | ____________ |
| Ethnic Group: | White ❑ / South Asian ❑ / Black ❑ / Other ❑ (specify) _____________________ | | |

| Phone Number / best way to contact, e.g. keyworkers: | | _____________________________________________ | |
| --- | --- | --- | --- |
| Type of Accom:  Current Accom Address (incl. postcode): | _____________________________  _____________________________  _____________________________  _____________________________ | Next of Kin:  Relationship:  Contact Tel No: | _____________________________  _____________________________  _____________________________ |

**Any registered GP?** Yes ❑ / No ❑

| GP Name:  GP Tel No:  GP Practice Code: | _____________________________  _____________________________  _____________________________ | GP Address: | ____________________________  ____________________________  ____________________________ |
| --- | --- | --- | --- |

**1 DEMOGRAPHICS** (continued)

**Overdose Information**

| Number of overdoses in past six months? | | _________ | Roughly, how many of your ODs needed somebody else to help, e.g. ambulance or accommodation staff? | | _________ |
| --- | --- | --- | --- | --- | --- |
| When was your most recent OD? | Date: _______________ | | Can you say why you overdosed? | _____________________  _____________________ | |
| What or who might stop you overdosing? | _____________________ | | Got Naloxone?  Know how to use it? | Yes ❑  Yes ❑ | No ❑  No ❑ |

**Known to Addictions Teams?** Yes ❑ / No ❑

| Addictions Team:  Worker’s Name: | _____________________________  _____________________________  _____________________________ | Addictions Team Address: | ____________________________  ____________________________  ____________________________  ____________________________ |
| --- | --- | --- | --- |

**Known to Mental Health Teams?** Yes ❑ / No ❑

| MH Team:  Worker’s Name: | _____________________________  _____________________________  _____________________________ | Mental Health Team Address: | ____________________________  ____________________________  ____________________________  ____________________________ |
| --- | --- | --- | --- |

| Any other teams/folk helping you? Yes ❑ / No ❑ | If yes, details: _________________________________  _____________________________________________ |
| --- | --- |

**Housing / Caseworker?** Yes ❑ / No ❑

| Housing Team:  Housing/Case- worker’s Name: | _____________________________  _____________________________  _____________________________ | Housing Team Address: | ____________________________  ____________________________  ____________________________  ____________________________ |
| --- | --- | --- | --- |

**Benefits**

| Currently applying for / appealing benefits decisions? | Yes ❑ | No ❑ | Description of benefits: | ____________________________  ____________________________ |
| --- | --- | --- | --- | --- |

**1 DEMOGRAPHICS** (continued)

**Travel**

| How do you usually travel to your GP / ADRS / MH / pharmacy, etc.? | | | _____________________________________________ | | |
| --- | --- | --- | --- | --- | --- |
| Do you have a bus pass / get taxis paid or what? | Yes ❑ | No ❑ | Does anybody usually go with you? | Yes ❑ | No ❑ |

**2 DIAGNOSES: PHYSICAL HEALTH**

| Patient reported: | From case notes (EMIS MH, EMIS GP, Portal, own GP): |
| --- | --- |
|  |  |

| Number of injection sites: | ________________ | | Location(s): | ___________________ | |
| --- | --- | --- | --- | --- | --- |
| Any wounds? | Yes ❑ | No ❑ | Wound Type(s):  _______________________  _______________________ | Wound(s) Location:  ___________________  ___________________ | |
| Other Skin Problems?  _______________________ | Yes ❑ | No ❑ | Dental Problems?  _______________________ | Yes ❑ | No ❑ |
| Seizures?  _______________________ | Yes ❑ | No ❑ | Allergies?  _______________________ | Yes ❑ | No ❑ |
| Eye Problems?  _______________________ | Yes ❑ | No ❑ | Hearing Problems?  _______________________ | Yes ❑ | No ❑ |
| Any broken bones?  _______________________ | Yes ❑ | No ❑ | Head injuries?  _______________________ | Yes ❑ | No ❑ |
| Assaulted in past 6 months?  _______________________ | Yes ❑ | No ❑ | Currently/recently pregnant?  _______________________ | Yes ❑ | No ❑ |

**3 DIAGNOSES: MENTAL HEALTH**

Any mental health problems (current or past):

| Patient reported: | From case notes (EMIS MH, EMIS GP, Portal, own GP): |
| --- | --- |
|  |  |

| Are you currently safe?  Yes ❑ / No ❑ | If no, details …  ___________________________________________________________________ |
| --- | --- |

**Depression Screen**

| Lost interest in things you used to enjoy? | Yes ❑ / No ❑ | Persistent low mood? | | Yes ❑ / No ❑ | |
| --- | --- | --- | --- | --- | --- |
| Any problems/feelings of: | | | | | |
| Sleep Increase ❑ | Sleep decrease ❑ | Activity increase ❑ | | Activity decrease ❑ | |
| Guilt/Worthlessness ❑ | Appetite Changes ❑ | Fatigue ❑ | | Concentration Poor ❑ | |
| For how long?   __________________ | Suicide attempts? Yes ❑ / No ❑ | Self-harm? Yes ❑ / No ❑ | | How are you feeling in yourself today?  _______________________ | |
| Over the last two weeks, how often have you been bothered by the following problems? | | | | | |
| 1. Feeling nervous, anxious or on edge 2. Not being able to stop/control worrying 3. Little interest or pleasure in doing things 4. Feeling down, depressed or hopeless | | Not at all  0  0  0  0 | Several days  1  1  1  1 | More than half the days  2  2  2  2 | Nearly every day  3  3  3  3 |

| Appearance: |  | Behaviour: |  |
| --- | --- | --- | --- |
|  |  |  |  |

**Problem Drug Use**

| Drug Name | Frequency | Last used | How much | Route | Since when |
| --- | --- | --- | --- | --- | --- |
| Heroin |  |  |  |  |  |
| Cocaine |  |  |  |  |  |
| Street Valium/Benzos |  |  |  |  |  |
| Spice |  |  |  |  |  |
| Gabapentin/Pregabalin |  |  |  |  |  |
| Cannabis |  |  |  |  |  |
| Other |  |  |  |  |  |

| Currently on methadone / buprenorphine / Buvidal / Espranor? | Yes ❑ No❑ | Dose: |
| --- | --- | --- |
| If not in treatment, have you ever been in treatment? | Yes ❑ No❑ | When and What?: |

**Problem Alcohol Use**

| Ever had a detox / hospital / rehab for alcohol? | | Yes ❑ | No ❑ | Previous DTs or withdrawals? | | Yes ❑ | No ❑ |
| --- | --- | --- | --- | --- | --- | --- | --- |
| Type of alcohol: ______________ | How much a day? ___________________ | | | | Age of first drink: ______ | | |

**5 LIFESTYLE**

**Smoking History**

| Current tobacco smoker? | Yes ❑ | No ❑ | Ex-Smoker (>10 years ago) | Yes ❑ | No ❑ |
| --- | --- | --- | --- | --- | --- |
| Non-smoker? | Yes ❑ | No ❑ | If current or ex-smoker, age started: | | ________ |
| No of cigarettes/day: | | ________ | No of Rollups/day: (half ounce = 15g tobacco = 20 cigarettes) | | ________ |

**Diet** – What do you eat in a typical day?

| Breakfast: | Lunch: | Dinner: |
| --- | --- | --- |

**Exercise in past four weeks**

| None | ❑ | Low (e.g. collecting prescription) | ❑ |
| --- | --- | --- | --- |
| Medium (e.g. walking) | ❑ | High (e.g. gym work) | ❑ |
| If you got the chance, what kind of work / activities would you want to do during the day? | | ______________________________________________ | |

**6 PRESCRIBED MEDICINES**

| **Patient Reported** | | **From Case Notes / Community Pharmacy** | | | **Picked > 80% in 3 months** |
| --- | --- | --- | --- | --- | --- |
| **Name and strength** | **Dose and frequency** | **Name and strength** | **Dose and frequency** | **Date started** |  |
|  |  |  |  |  |  |
|  |  |  |  |  |  |
|  |  |  |  |  |  |
|  |  |  |  |  |  |
|  |  |  |  |  |  |
|  |  |  |  |  |  |
|  |  |  |  |  |  |

**7 HEALTH MEASURES**

| Height (cm): _______ | Weight (kg): _______ | BMI: _______ |  |
| --- | --- | --- | --- |
| Compared with one year ago, has your weight changed? | Yes ❑ / No ❑ | Lost weight ❑ | Gained weight: ❑ |
| Blood Pressure (mmHg): |  | Heart Rate / Pulse: |  |
| O2 Sats (%): |  | Grip Strength: |  |
| PEFR: | …….... …..….. …….... % predicted (<30-50% = severe/acute) | | |
| How would you describe your usual walking pace? | | Slow ❑ | Other ❑ |

In terms of your breathing …

| Are you too breathless to leave your accommodation or breathless when getting dressed? | Yes ❑ | No ❑ |
| --- | --- | --- |
| Do you have to stop for a breath after walking 100 yards on the flat or after a few mins? | Yes ❑ | No ❑ |
| Do you walk slower than people who are ages with you because of breathlessness or do you have to stop for breath when walking at your own pace? | Yes ❑ | No ❑ |
| Are you breathless when in a rush or walking up a slight hill? | Yes ❑ | No ❑ |
| Do you get breathless only with hard exercise? | Yes ❑ | No ❑ |

| Over the past two weeks, how often have you felt tired or had little energy? | More than ❑ half the days | Nearly ❑ every day | Other ❑ |
| --- | --- | --- | --- |

| Mother or father, brother or sister ever suffered any of the following while <60 years old? | MI ❑ | Angina ❑ | TIA ❑ | Stroke ❑ |
| --- | --- | --- | --- | --- |

**8 QUALITY OF LIFE**

| We would like to know how good or bad your health is TODAY  100 means the best health you can imagine. 0 means the worst health you can imagine |
| --- |

| Under each heading, please tick the ONE box that best describes your health TODAY.  10  0  20  30  40  50  60  80  70  90  100  5  15  25  35  45  55  75  65  85  95 | |
| --- | --- |
| **MOBILITY** |  |
| I have no problems in walking about | ❑ |
| I have slight problems in walking about | ❑ |
| I have moderate problems in walking about | ❑ |
| I have severe problems in walking about | ❑ |
| I am unable to walk about | ❑ |
| **SELF-CARE** |  |
| I have no problems washing or dressing myself | ❑ |
| I have slight problems washing or dressing myself | ❑ |
| I have moderate problems washing or dressing myself | ❑ |
| I have severe problems washing or dressing myself | ❑ |
| I am unable to wash or dress myself | ❑ |
| **USUAL ACTIVITIES** *(e.g. work, study, housework, family or leisure activities)* |  |
| I have no problems doing my usual activities | ❑ |
| I have slight problems doing my usual activities | ❑ |
| I have moderate problems doing my usual activities | ❑ |
| I have severe problems doing my usual activities | ❑ |
| I am unable to do my usual activities | ❑ |
| **PAIN / DISCOMFORT** |  |
| I have no pain or discomfort | ❑ |
| I have slight pain or discomfort | ❑ |
| I have moderate pain or discomfort | ❑ |
| I have severe pain or discomfort | ❑ |
| I have extreme pain or discomfort | ❑ |
| **ANXIETY / DEPRESSION** |  |
| I am not anxious or depressed | ❑ |
| I am slightly anxious or depressed | ❑ |
| I am moderately anxious or depressed | ❑ |
| I am severely anxious or depressed | ❑ |
| I am extremely anxious or depressed | ❑ |

Your Health Number Today is: ______

How do you think you could improve this number? ______________________________________

**9 EXPERIENCE WITH TREATMENT & SELF MANAGEMENT**

Questions available on request.

**DATA FROM CASE NOTES, AFTER INTERVIEW …**

**Healthcare contacts in past six months (from clinical records: EMIS GP, EMIS MH, PORTAL …)**

| **Primary Care** | **Number of Contacts** (S = Scheduled / U = Unscheduled) | **Dates and Durations** |
| --- | --- | --- |
| GP |  |  |
| Pharmacist |  |  |
| Nurse |  |  |
| Other |  |  |
| **Addictions** |  |  |
| Social Care Worker |  |  |
| AHP |  |  |
| Nursing |  |  |
| Pharmacist |  |  |
| Medical |  |  |
| Psychiatrist |  |  |
| Other … |  |  |
| **Mental Health** |  |  |
| Psychiatrist |  |  |
| MH Nurse |  |  |
| MH Worker |  |  |
| **Hospital** |  |  |
| ED |  |  |
| Hospitalisations |  |  |
| Outpatient (specify type) |  |  |
| Mental Health |  |  |

**Social Work Contacts**

| **Housing** | **Number of Contacts** | **Dates** |
| --- | --- | --- |
|  |  |  |
|  |  |  |
| **Third Sector** |  |  |
|  |  |  |
|  |  |  |

**Rehab Contacts**

| **Type & Name** | **Number of Contacts** | **Dates and Durations** |
| --- | --- | --- |
|  |  |  |
|  |  |  |

**BLOODS (most recent, in past year)**

| **Type** | **Normal or not … if not what? Record actual results, taking closest to recruitment except for Magnesium (take lowest in preceding year)** | **Date** |
| --- | --- | --- |

**Urea & Electrolytes**

| Sodium |  |  |
| --- | --- | --- |
| Potassium |  |  |
| Chloride |  |  |
| Creatinine |  |  |
| eGFR |  |  |

**Liver Function Tests**

| ALT |  |  |
| --- | --- | --- |
| AST |  |  |
| ALP |  |  |
| Albumin |  |  |

**Bone Profile**

| Calcium |  |  |
| --- | --- | --- |
| Calcium (adjusted) |  |  |
| Phosphate |  |  |

| CRP |  |  |
| --- | --- | --- |
| B12 |  |  |
| RCC |  |  |
| Magnesium |  |  |
| Folate |  |  |
| Cholesterol |  |  |
| PFTs |  |  |

**BBVs**

| HCV |  |  |
| --- | --- | --- |
| HIV |  |  |

| **Thanks and voucher given?** (£10 baseline interview, £10 subsequent interviews – 6/9 months post) | ❑ |  |
| --- | --- | --- |
| Time taken for interview | ______ hr(s) _______ mins | |
| Phone for randomisation | ❑ |  |
| Allocation | ACTIVE ❑ | CONTROL ❑ |

| Requested access to look up of clinical notes | ❑ |  |
| --- | --- | --- |
| Add Alert to EMIS Web | ❑ |  |

| Baseline Form passed back to office: | Date: ____________ |
| --- | --- |
| Data entered onto spreadsheet: | Date: ____________ / By Whom: ____________ |
| Checked on: | Date: ____________ / By Whom: ____________ |
